# Physical activity behavior in the first month after mild traumatic brain injury is associated with physiological and psychological risk factors for chronic pain

**DOI:** 10.1097/PR9.0000000000000969

**Published:** 2021-10-29

**Authors:** Kelly M. Naugle, Sam Corrona, Jared A. Smith, Tyler Nguyen, Jonathan Saxe, Fletcher A. White

**Affiliations:** a Department of Kinesiology, School of Health and Human Sciences, Indiana University Purdue University Indianapolis, Indianapolis, IN, USA; b Medical Scientist Training Program, Indiana University School of Medicine, Indianapolis, IN, USA; c Department of Anesthesia, Indiana University School of Medicine, Indianapolis, IN, USA; d Trauma Department, Ascension St. Vincent Indianapolis Hospital, Indianapolis, IN, USA; e Richard L. Roudebush Veterans Medical Center, Indianapolis, IN, USA

**Keywords:** Conditioned pain modulation, Physical activity, Mild traumatic brain injury, Pain catastrophizing, Pain modulation

## Abstract

**Objective:**

The purpose of this study was to determine whether self-reported physical activity (PA) in the first month after mild traumatic brain injury (mTBI) predicts endogenous pain modulatory function and pain catastrophizing at 1 to 2 weeks and 1 month after injury in patients with mTBI.

**Methods:**

Patients with mild traumatic brain injury completed study sessions at 1 to 2 weeks and 1 month after injury. Assessments included a headache survey, Pain Catastrophizing Scale, International Physical Activity Questionnaire-Short Form, and several quantitative sensory tests to measure endogenous pain modulatory function including conditioned pain modulation (CPM), temporal summation, and pressure pain thresholds of the head. Hierarchical linear regressions determined the relationship between the PA variables (predictors) and pain catastrophizing and pain modulation variables (dependent variables) cross-sectionally and longitudinally, while controlling for potential covariates.

**Results:**

In separate hierarchical regression models, moderate PA, walking, and total PA at 1 to 2 weeks after injury predicted pain inhibition on the CPM test at 1 month, after controlling for significant covariates. In addition, a separate regression revealed that minutes sitting at 1 month predicted CPM at 1 month. Regarding predicting pain catastrophizing, the regression results showed that sitting at 1 to 2 weeks after injury significantly predicted pain catastrophizing at 1 month after injury.

**Conclusion:**

Greater self-reported PA, especially moderate PA, 1 to 2 weeks after injury longitudinally predicted greater pain inhibitory capacity on the CPM test at 1 month after injury in patients with mTBI. In addition, greater sedentary behavior was associated with worse pain inhibition on the CPM test and greater pain catastrophizing at 1 month after injury.

## Abstract 2

Lower levels of physical activity in the first month after mild TBI predicts decreased conditioned pain modulation and greater pain catastrophizing in patients with mild TBI.

## 1. Introduction

Approximately 1.7 million traumatic brain injuries (TBIs) occur in adults each year in the United States, with mild TBIs (mTBIs) accounting for most (∼79%) of these injuries.^20^ The early management of mTBI (0–7 days after injury) typically includes recommendations of physical rest during recovery (ie, symptom resolution),^13,33^ although little evidence supports the use of physical rest to improve mTBI outcomes. Importantly, emerging research indicates that subsymptom threshold physical activity (PA) introduced after the acute recovery period seems safe and that prolonged periods of physical rest after injury may be detrimental to physical and psychological recovery up to 1 month after mTBI.^22,24^ However, the body of literature evaluating relationships of PA behavior with mTBI outcomes is mixed and extremely limited. Most of these studies are conducted in athletes, adolescents, and children. Furthermore, this literature has rarely considered the impact of early PA patterns post-mTBI on pain-related outcomes, although pain is one of the most common and persistent symptoms after mTBI, especially posttraumatic headaches.

In civilian adult populations, persistent posttraumatic headaches develop in approximately half of those who have had a mTBI.^15,28,34^ The mechanisms underlying the development of persistent posttraumatic headaches are clearly multifaceted but remain uncertain. Mild traumatic brain injury is often accompanied by psychological distress,^32^ including anxiety, depression, and pain catastrophizing.^3^ In general, a substantial amount of evidence supports the importance of these psychosocial factors in shaping pain-related experiences, including PTH.^1,5,9,14^ Also, recent studies suggest abnormal and unbalanced endogenous pain modulatory function (ie, decreased pain inhibitory function and enhanced sensitization on quantitative sensory tests [QSTs]) early after the injury may play a role in facilitating persistent posttraumatic headache development.^35,38^ For example, our previous longitudinal study revealed that decreased endogenous pain inhibition on the conditioned pain modulation (CPM) test within the first month of injury predicted the presence of posttraumatic headaches 4 months postinjury in patients with mTBI.^35^ Notably, a growing body of evidence in non-TBI populations has begun to link PA behavior to endogenous pain modulatory function as measured by QSTs^10,36,37,40^ and pain catastrophizing,^46^ with generally more efficacious pain modulation and less pain catastrophizing associated with greater PA levels. However, the relationship between endogenous pain modulatory function, pain catastrophizing, and PA behavior early after mTBI is unknown.

Thus, the purpose of this prospective, observational study was 2-fold. We sought to determine whether self-reported PA early after a mTBI predicts (1) endogenous pain modulatory function as measured by QSTs and (2) pain catastrophizing in adults with mTBI within the first month after injury. Tests of pain modulatory function by QST, headache pain, pain catastrophizing, and self-reported PA were measured at 1 to 2 weeks and 1 month after injury. We hypothesized that patients with mTBI who did greater amounts of PA in the first month after injury would exhibit more efficacious pain modulation and less pain catastrophizing.

## 2. Methods

### 2.1. Participants

Adults (18–65 years of age) with mTBI were enrolled in this study. Mild traumatic brain injury participants had to have a mTBI diagnosis according to the criteria recommended by the World Health Organization Task Force.^4^ The mTBI could not be due to drugs, alcohol, medications, caused by other injuries or treatments for other injuries, caused by other problems (ie, coexisting medical conditions and psychological trauma), or a penetrating craniocerebral injury. Exclusion criteria included the following: (1) chronic cardiovascular disease or uncontrolled hypertension, (2) metabolic disease, (3) neurological disease, (4) serious psychiatric conditions or hospitalization within the preceding year for psychiatric illness, (5) chronic headaches before the head injury, (6) current involvement in litigation, (7) chronic use of narcotics, and (8) fracture or polytrauma at the time of head injury. These data are part of a larger study evaluated risk factors for persistent posttraumatic headache.^3,35^

#### 2.1.1. Recruitment

Mild traumatic brain injury participants were recruited from level 1 trauma centers within hospitals located in the Indianapolis area. Potentially eligible patients had their electronic medical records screened by study recruiters to identify patients who met the inclusion or exclusion criteria. Mild traumatic brain injury diagnosis was also confirmed by the attending emergency department (ED) physician. If the patient expressed interest in the research, his or her identification was put into a secure database. Then, research staff would try to contact the potential participant within 48 hours to try to schedule the laboratory session within 2 weeks of the injury.

### 2.2. Procedures

The Indiana University and Ascension St. Vincent Hospital Human Subject Review Boards approved this study. All the participants used in this study completed one study visit occurring within 2 weeks after injury and a second visit occurring approximately 1 month after injury. During visit 1, participants reviewed and signed a written institutional review board-approved informed consent form. To verify participants who met the inclusion or exclusion criteria, participants completed a health history questionnaire supplemented by an interview. During each visit, participants completed the same questionnaires and QSTs. These assessments are described below. All participants were asked to refrain from pain-relief medication and consuming caffeine on the day of testing before their session.

### 2.3. Outcome measures

#### 2.3.1. Questionnaires

##### 2.3.1.1. International Physical Activity Questionnaire-Short Form

Self-reported PA was measured with the International Physical Activity Questionnaire-Short Form (IPAQ-SF). The IPAQ-SF asks subjects to recall the amount of time during the past 7 days spent in vigorous activities, moderate activities, walking, and sitting.^7,8^ Guidelines provided by www.ipaq.ki.se were used for data processing and scoring of the questionnaire. Scores were calculated in terms of Met-minutes or week for vigorous activity, moderate activity, walking, and total activity. The Sitting subscale represents the number of minutes reported sitting per week. The IPAQ-SF has shown acceptable concurrent and construct validity and test–retest reliability (0.66–0.89).^7,23^

##### 2.3.1.2. Pain Catastrophizing Scale

The Pain Catastrophizing Scale (PCS) assesses negative mental responses to anticipated or actual pain.^39^ The PCS has 13 items that are scored on a Likert scale with 3 subcategories: rumination, magnification, and helplessness. Scores range from 0 to 52, with higher PCS scores indicative of higher pain catastrophizing.

##### 2.3.1.3. Headache survey

A headache survey that has been used successfully in previous studies of posttraumatic headache was administered to all patients.^28,29^ The survey included questions about ongoing headache (frequency, intensity, duration, medication use, triggers, and other treatments), history of problems with headache preinjury, and characteristics of ongoing headache (headache symptoms). Participants rated the average pain intensity of their headaches during the past week using a 0 to 10 Numerical Rating Scale (NRS), with 0 being no headaches at all and 10 being the worst pain possible.

#### 2.3.2. Measures of pain modulatory function

Before each QST test, subjects were made familiar with each sensory test to be performed and were taught the 0 to 100 pain rating system. The tests of sensitization (pressure pain sensitivity and temporal summation [TS] of pain) were performed first followed by the CPM test. A minimum of 10 minutes separated each sensitization and CPM test.

##### 2.3.2.1. Pain sensitization measures

Several QSTs in human experimental studies have been used to identify the presence of pain sensitization including TS of pain and generalized pressure sensitivity.^41^

###### 2.3.2.1.1. Pressure pain sensitivity of the head or neck area

Pressure pain thresholds (PPTs) were tested on the following 5 sites of the head and neck areas, as has been conducted in prior research^11^: (1) middle of the forehead, (2) left temple, (3) parietal area (top of head), (4) posterior neck/C2, and (5) left trapezius. A digital, handheld, clinical grade pressure algometer (AlgoMed; Medoc Advanced Medical Systems, Durham, NC) with a 1.0 cm^2^ probe was placed against the skin of one of the 5 sites, and pressure was gradually increased at a slow constant rate of pressure (30 kPA/s). The participant was instructed to verbally signal when (s)he first experienced pain caused by the pressure device at which time the algometer was removed. Two trials were performed at each site with 20-second intervals between each trial. The PPTs at all sites were averaged for a single PPT score (PPT-Head) to be used in data analysis.^11^

###### 2.3.2.1.2. Mechanical temporal summation

Temporal summation is an indirect method of evaluating hyperexcitability of the central nervous system.^41^ Mechanical temporal summation (MTS) was tested on the back of the hand and the middle of the forehead using the von Frey filament (Touch Test Sensory Evaluator 6.65) calibrated to bend at 300 g of pressure. First, a single pinprick was applied with the filament to the body site. Participants rated the perceived pain intensity using a NRS of 0 (no pain at all) to 100 (worst pain imaginable). Then, a series of 10 pinprick stimuli using the same monofilament was applied to the body site within an area of 1 cm^2^ and at a rate of 1 tap per second. Participants were asked to immediately rate the greatest pain intensity experienced during the 10 pinprick stimuli using the 0 to 100 NRS. The TS value was calculated as the difference between the pain rating after the 10 stimuli and the first stimuli. This procedure was repeated twice at each body site with a 60-second rest interval between trials. The 2 trials at each site were averaged for a single MTS hand and MTS forehead score.

##### 2.3.2.2. Conditioned pain modulation

The most frequently used test of endogenous pain inhibition in humans is CPM. Conditioned pain modulation refers to the reduction of pain produced by a test stimulus by a second noxious conditioning stimulus in a remote body site (ie, “pain inhibition by pain”).^42,43^ For the CPM test, PPTs (test stimulus) on the left arm were measured before and immediately after the submersion of the right hand in a cold water bath (conditioning stimulus). Seven minutes separated the pre-PPT trials and the initiation of the conditioning stimulus, during which the participants sat quietly. This period of rest was included to prevent within-session adaptation.^21^ *Test stimulus*: The test stimulus was PPTs administered on the left volar forearm, using the same PPT threshold procedures as described above (except different body location). Two trials were administered consecutively during each preconditioning and postconditioning test. The posttest trials were administered immediately after participants removed their hand from the cold-water bath. These trials were averaged for a single pretest and post-test PPT score. *Conditioning stimulus*: Participants immersed their right hand up to the wrist in a cold-water bath (VersaCool 7; Thermo Scientific, Waltham, MA) maintained at 10°C for 1 minute. Cold pain was assessed every 15 second using the 0 to 100 NRS. The pain ratings were averaged across time for a single cold-water immersion pain score for each participant. *Calculation of CPM*: A percent change score was calculated for the test stimulus with the following formula: [(post PPT trial score−pre PPT trial score)/pre PPT trial score]∗100. A positive percent change score indicated an increase in PPTs following the conditioning stimulus and thus pain inhibition.

### 2.4. Statistical analysis

Descriptive statistics were calculated for all the outcome variables. The Shapiro–Wilk test of normality indicated that all variables were not normally distributed. Thus, Mann–Whitney *U* tests were conducted to determine if outcome variables differed by sex and pain medication status (taking pain meds vs no pain meds). Wilcoxon signed-rank tests were conducted to compare measures between time points (1 week vs 1 month). We conducted spearman bivariate correlations between age, headache pain intensity, and the primary outcome variables at the 1 to 2 week and 1-month time points. The target *P*-value was set at 0.05, with Holm–Bonferroni corrections applied to deal with familywise error rates for multiple hypothesis tests.^16^ In addition, hierarchical linear regressions were performed to determine the relationship between the PA variables (predictors) and pain catastrophizing and pain modulation variables (dependent variables) cross-sectionally (1-week PA predicting 1-week pain modulation and 1-month PA predicting 1-month pain modulation) and longitudinally (1-week PA predicting 1-month pain modulation), while controlling for potential covariates. Regression analysis was only conducted between variables if a correlation existed between the primary independent and dependent variable at *P* < 0.05. Potential covariates were chosen from the correlational analyses. Only significant covariates were retained for the final regression models. International Physical Activity Questionnaire variables were always entered into the last block for each regression. The *P*-value for significance was *P* < 0.05.

## 3. Results

### 3.1. Participants

A power analysis using G*Power 3.0.10 was used to estimate the sample size needed for predicting the change in *R*^2^ in a multiple linear regression model, when the independent variable of interest was added to the model. With an estimated moderate effect size (f^2^ = 0.15) and including 2 covariates, a sample size of 55 participants would provide power of 0.80 at alpha = 0.05. Seventy-four participants enrolled in this study. Seven participants dropped out before completing visit 2. Based on International Physical Activity Questionnaire (IPAQ) data processing rules, cases in which the sum of all PA subscales were greater than 960 minutes were excluded from the analyses (n = 6). Some participants answered “don't know or not sure” for questions on the IPAQ; thus, totals for these scales could not be computed. Thus, final sample size for data analyses ranged from 57 to 61.

Participant characteristics are presented in Table 1. GCS scores ranged from 14 to 15. Causes of mTBI included hit by a vehicle (n = 4), hit by a object on the head (n = 8), fight (n = 2), vehicle accident (n = 24), and fall (n = 18). Men reported greater walking at 1 to 2 weeks (*P* = 0.044), vigorous PA at 1 month (*P* = 0.026), and PPTs at the head at 1 month (*P* = 0.002) compared with women. Most participants reported taking pain medications for headaches at 1 to 2 weeks postinjury. As expected, those taking pain medications at 1 to 2 weeks after injury had greater headache pain intensity compared with those not taking medication at 1 to 2 weeks, *P* = 0.015. At 1 month after injury, just over half of participants were still taking medications for headache pain. Headache pain intensity at 1 to 2 weeks (*P* < 0.001) and 1 month (*P* < 0.001) and pain catastrophizing at 1 to 2 weeks (*P* = 0.047) and at 1 month (*P* = 0.006) were greater, and vigorous PA at 1 to 2 weeks was lower (*P* = 0.016) in those taking pain medication at 1 month compared with those not taking pain medication at 1 month. The Wilcoxon signed-rank tests showed that headache pain intensity, pressure pain sensitivity, and PCS scores decreased, whereas PA scores increased from 1 week to 1 month after injury (Table 1).

**Table 1:** Participant characteristics (n = 61).

Variable	1–2 wk postinjury	1 mo postinjury	*P*
Age, y	32.9 ± 9.2		
Sex, % female	50.8		
Education, %			
Some HS	9.8		
HS degree	36.1		
2 y college degree	19.7		
4 y college degree	13.1		
Masters or above	18.0		
Race, %			
African American	24.6		
Asian/Pacific Islander	3.3		
Anglo-American	55.7		
Hispanic	8.2		
Others	8.2		
Percent reporting LOC with mTBI	44.3		
Percent reporting headaches	90	78.7	
Percent taking pain medication	80.3	59	
Average headache pain intensity (0–10 scale)	6.0 (3.5 to 7)	5.0 (3 to 7)	0.002
Conditioned pain modulation, % change	15.7 (−1.2 to 26.9)	21.4 (2.8 to 37.7)	0.139
PPT-head, kPA	196.2 (143 to 298)	220.0 (157 to 340)	0.014
TS—forehead	8.0 (2.3 to 18.8)	10.0 (2.3 to 15.5)	0.716
TS—hand	5.0 (0 to 14)	6.5 (0.25 to 15)	0.928
Pain Catastrophizing Scale score	17.0 (8.5 to 25.5)	9.0 (3 to 23)	0.002
IPAQ-SF data, Met-minutes/week			
Vigorous PA	0.0 (0 to 600)	1080.0 (180 to 3780)	<0.001
Moderate PA	80.0 (0 to 690)	900.0 (420 to 4800)	<0.001
Walking	693.0 (182 to 2772)	1980 (495 to 6757)	0.002
Total PA	1611.0 (564 to 5885)	4893.0 (1803 to 15,388)	<0.001
Sitting, minutes per wk	2100.0 (1120 to 4200)	2100 (1803 to 15,388)	0.992

Data presented as percentages or median (interquartile range). *P*-values are from the results of the Wilcoxon signed-rank test comparing 1-week values to 1-month values.

HS, high school; IPAQ-SF, International Physical Activity Questionnaire-Short Form; LOC, loss of consciousness; mTBI, mild traumatic brain injur; PA, physical activity; PPT, pressure pain threshold; TS, temporal summation.

### 3.2. Spearman rank correlations

**Figure 1.**
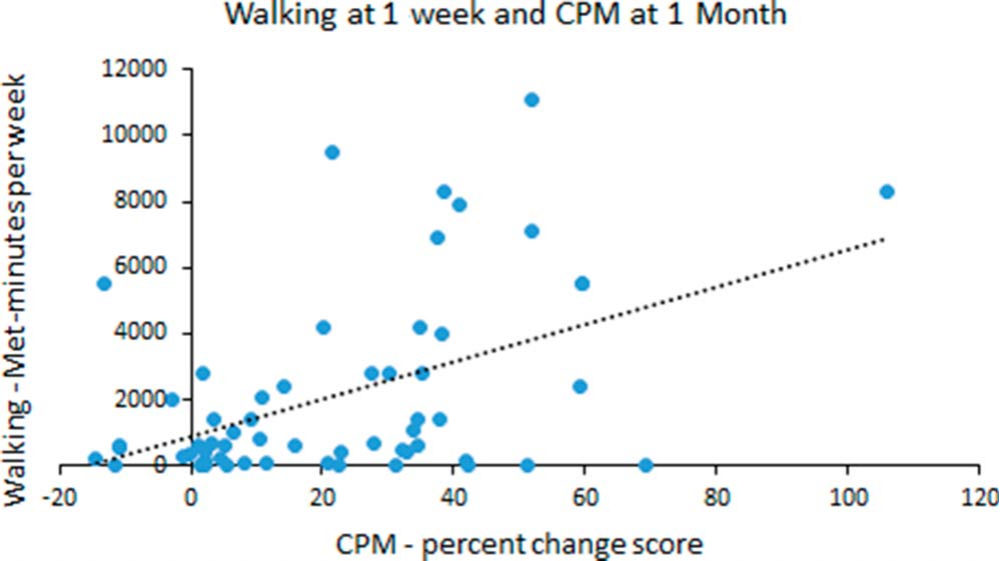
Scatter plot of the relationship between the walking subscale score of the International Physical Activity Questionnaire-Short Form at 1 to 2 weeks after injury and CPM score at 1 month after injury. CPM, conditioned pain modulation.

**Figure 2.**
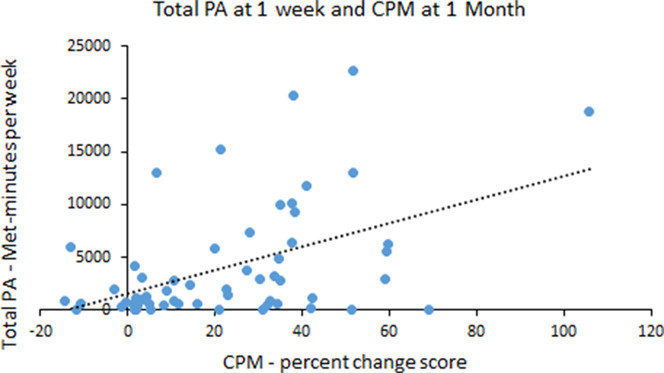
Scatter plot of the relationship between the total PA International Physical Activity Questionnaire-Short Form score at 1 to 2 weeks after injury and CPM score at 1 month after injury. CPM, conditioned pain modulation; PA, physical activity.

Table 2 shows the correlation coefficients between the primary outcome measures at 1 to 2 weeks after injury. Total PA and PCS were correlated at *P* < 0.05. However, this relationship was not significant after Holm–Bonferroni corrections were applied. Table 3 shows the correlation coefficients between the primary outcome measures at 1 month after injury. After the Holm–Bonferroni corrections, no significant correlations existed between the PA and pain modulation variables. Pain catastrophizing was positively correlated with intensity of headache pain (*r* = 0.506, *P* < 0.001). Table 4 shows the correlation coefficients between the PA variables at 1 to 2 weeks after injury with the pain modulation variables and PCS at 1 month after injury. After the Holm–Bonferroni corrections, greater inhibition on the CPM test at 1 month was significantly related to greater walking (Fig. 1 for the scatter plot) and total PA (Fig. 2 for the scatter plot) at 1 to 2 weeks after injury. Conditioned pain modulation was also negatively correlated with headache pain intensity at 1 to 2 weeks after injury (*r* = −0.314, *P* = 0.014). Age was not significantly correlated with any variables.

**Table 2:** Correlation coefficients for the association of physical activity variables at 1–2 weeks with conditioned pain modulation, temporal summation, pressure pain threshold, and Pain Catastrophizing Scale at 1–2 weeks postinjury.

IAPQ variable	CPM	TS-hand	TS-forehead	PPT-head	PCS
Vigorous PA	−0.073	−0.044	0.049	0.136	−0.189
Moderate PA	0.079	−0.070	−0.100	0.130	−0.171
Walking	0.199	−0.012	0.037	0.049	−0.179
Total PA	0.053	−0.001	0.043	0.088	−0.287*
Sitting	0.016	−0.117	−0.129	0.117	0.180

* Significant *P* < 0.05 with no correction.

†Significant after Holm–Bonferroni correction.

CPM, conditioned pain modulation; IAPQ, International Physical Activity Questionnaire; PA, physical activity; PCS, Pain Catastrophizing Scale; PPT, pressure pain threshold; TS, temporal summation.

**Table 3:** Correlation coefficients for the association of physical activity variables at 1 month with conditioned pain modulation, temporal summation, pressure pain threshold, and Pain Catastrophizing Scale at 1 month postinjury.

IAPQ variable	CPM	TS-hand	TS-forehead	PPT-head	PCS
Vigorous PA	0.015	0.087	0.146	0.080	0.001
Moderate PA	0.218	−0.041	−0.010	0.246	−0.057
Walking	0.106	−0.239	−0.177	0.270*	0.032
Total PA	0.158	−0.092	−0.013	0.126	−0.017
Sitting	−0.280*	−0.040	−0.143	0.124	0.115

* Significant *P* < 0.05 with no correction.

†Significant after Holm–Bonferroni correction.

CPM, conditioned pain modulation; IAPQ, International Physical Activity Questionnaire; PA, physical activity; PCS, Pain Catastrophizing Scale; PPT, pressure pain threshold; TS, temporal summation.

**Table 4:** Correlation coefficients for the association of physical activity variables at 1–2 weeks with conditioned pain modulation, temporal summation, pressure pain threshold, and Pain Catastrophizing Scale at 1 month postinjury.

IAPQ variable	CPM	TS-hand	TS-forehead	PPT-head	PCS
Vigorous PA	0.160	−0.027	0.081	0.148	−0.193
Moderate PA	0.263*	0.076	0.013	0.064	−0.154
Walking	0.356†	−0.018	−0.051	0.015	−0.170
Total PA	0.410†	−0.066	−0.057	0.080	−0.269*
Sitting	−0.262*	−0.061	−0.235	0.151	0.262*

* Significant *P* < 0.05 with no correction.

† Significant after Holm–Bonferroni correction.

CPM, conditioned pain modulation; IAPQ, International Physical Activity Questionnaire; PA, physical activity; PCS, Pain Catastrophizing Scale; PPT, pressure pain threshold; TS, temporal summation.

### 3.3., Hierarchical linear regression analyses

#### 3.3.1. Pain modulation variables

The TS variables were not associated with any PA variables and thus were not further investigated with linear regression analyses. The only variable correlated with PPTs of the head (1 month) at the *P* < 0.05 level was walking (1-month); however, the regression model was not significant, *P* = 0.327.

Conditioned pain modulation at 1 to 2 weeks was not correlated with any IPAQ variables at 1 to 2 weeks at the *P* < 0.05 level, and therefore, these relationships were not further investigated with linear regression analyses. However, several PA variables correlated with CPM at 1 month at the *P* < 0.05 level. Four separate longitudinal regression models were conducted with CPM at 1 month as the dependent variable and either moderate PA, walking, total PA, or sitting as the final predictor. In each model, headache pain intensity at 1 to 2 weeks was added in step 1. The PA variable was added in step 2. The model with sitting as the final predictor was significant (*P* = 0.017); however, sitting at 1 to 2 weeks did not significantly predict CPM at 1 month (Beta = −0.176, *P* = 0.167). The regression models with moderate PA, walking, or total PA at 1 to 2 weeks as the final predictor were significant. The regression model accounting for the highest variance in CPM at 1 month was with total PA at 1 to 2 weeks as the final predictor. The results showed that greater PA at 1 to 2 weeks after injury predicted greater pain inhibitory capacity on the CPM test at 1 month after injury in the patients with mTBI. Based on the correlation results, we also evaluated whether sitting at 1 month predicted CPM at 1 month. The overall model was significant, with greater sitting at 1 month predicting lower pain inhibition on the CPM test at 1 month after injury. The regression results from these analyses are presented in Table 5.

**Table 5:** Summary of significant hierarchical regression models for conditioned pain modulation at 1 month postinjury with physical activity variables as final predictors.

A. Walking at 1–2 wk predicting CPM at 1-mo (n = 58)					
Step variables	Δ*R*^2^	Standardized β	Unstandardized B	*P* for β	Model *P*
Headache pain 1–2 wk	0.099	−0.128	−1.15	0.336	0.001
Walking 1–2 wk	0.140	0.419	0.004	0.002	

B. Moderate PA at 1–2 wk predicting CPM at 1-mo (n = 60)					
Step variables	Δ*R*^2^	Standardized β	Unstandardized B	*P* for β	Model *P*
Headache pain 1–2 wk	0.099	−0.227	−2.60	0.024	0.002
Moderate PA 1–2 wk	0.102	0.321	0.005	0.009	

C. Total PA at 1–2 wk predicting CPM at 1 mo (n = 58)					
Step variables	Δ*R*^2^	Standardized β	Unstandardized B	*P* for β	Model *P*
Headache pain 1–2 wk	0.099	−0.154	−1.39	0.227	<0.001
Total PA 1–2 wk	0.153	0.422	0.002	0.001	

D. Sitting at 1 mo predicting CPM at 1 mo (n = 59)					
Step variables	Δ*R*^2^	Standardized β	Unstandardized B	*P* for β	Model *P*
Headache pain 1–2 wk	0.101	−0.303	−2.83	0.016	0.005
Sitting 1 mo	0.072	−0.268	−0.004	0.032	

CPM, conditioned pain modulation; PA, physical activity.

#### 3.3.2. Pain Catastrophizing Scale

**Figure 3.**
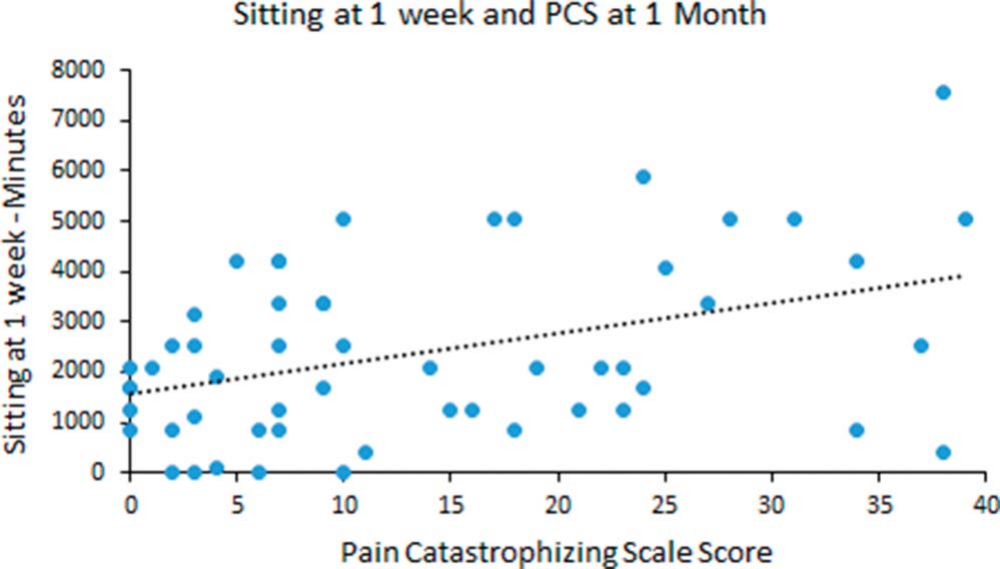
Scatter plot of the relationship between the sitting subscale score of the International Physical Activity Questionnaire-Short Form at 1 to 2 weeks after injury and PCS score at 1 month after injury. PCS, Pain Catastrophizing Scale.

Total PA at 1 to 2 weeks was the only variable correlated with PCS at 1 to 2 weeks at the *P* < 0.05 level; therefore, we conducted a regression with total PA as the predictor of PCS at 1 to 2 weeks. This regression model was not significant, *P* = 0.081. The PA variables at 1 month were not associated with PCS at 1 month, and thus, these relationships were not further investigated with linear regression analyses. However, sitting and total PA at 1 to 2 weeks were correlated with PCS at 1 month at the *P* < 0.05 level. Initial variables considered in the regression model with PCS at 1 month as the dependent variable included headache pain intensity at 1 month, pain medication status at 1 month, sitting at 1 to 2 weeks, and total PA at 1 to 2 weeks. As shown in Table 6, the final model included only headache pain intensity in step 1 and sitting in step 2. Sitting at 1 to 2 weeks after injury significantly predicted pain catastrophizing at 1 month after injury, with greater sitting related to worse pain catastrophizing (Fig. 3 for the scatter plot of sitting and PCS).

**Table 6:** Sitting at 1–2 weeks predicting the Pain Catastrophizing Scale at 1 month.

Step variables	Δ*R*^2^	Standardized β	Unstandardized B	*P* for β	Model *P*
Headache pain 1 mo	0.192	0.415	1.68	0.001	<0.001
Sitting 1–2 wk	0.072	0.269	0.001	0.026	

## 4. Discussion

The effects of PA behavior on pain-related outcomes after mTBI have not been widely investigated. This study provides the first evidence suggesting that PA behavior in the first month after a mTBI is related to the functioning of the endogenous pain modulatory systems, as well as pain-related psychological processes in patients with mTBI. Generally, greater PA was associated with better outcomes. These findings challenge the long-held practice that physical rest in the early stages after injury is optimal for physiological and psychological recovery.

Recent evidence has identified deficient endogenous pain inhibition on the CPM test as a risk factor for the development of chronic pain after mTBI.^35,38^ Importantly, our results suggest that self-reported PA levels early after mTBI are strong predictors of pain inhibitory function on the CPM test in patients with mTBI, even after controlling for potential covariates. Specifically, patients with mTBI who reported greater total PA 1 to 2 weeks after injury exhibited greater pain inhibition on the CPM test at 1 month after injury. This relationship seemed to be driven by the moderate PA and walking IPAQ subscales vs the vigorous PA subscale. In addition, individuals reporting greater sedentary behavior at 1 month also exhibited decreased pain inhibitory capacity. Previous cross-sectional research has shown that pain inhibitory function is related to PA behavior in healthy younger adults,^37,40^ older adults,^36^ triathletes,^12^ and fibromyalgia patients.^10^ Indeed, studies in younger adults have revealed links between vigorous PA and more efficient CPM,^37,40^ whereas studies in older adults and individuals with fibromyalgia have demonstrated relationships between pain inhibitory function and light PA or sedentary behavior.^10,36^ Our study is one of the first to provide longitudinal evidence that PA behavior can influence endogenous pain inhibition in adults with mTBI.

The mechanisms through which PA could influence pain inhibitory function after mTBI are not clear. The brain circuitry regulating CPM lies within the brainstem and is greatly dependent on descending serotonergic pathways.^6,25,45^ Animal research has shown increased availability of serotonin in the CNS with low-intensity PA^2^ and enhanced expression of serotonin receptors that participate in pain modulation with treadmill exercise.^27^ Conditioned pain modulation circuitry also involves modulation by higher cortical regions, such as the cingulate and prefrontal cortices.^44^ Liu et al^26^ recently demonstrated that different types of exercise can modulate the resting functional connectivity between regions of the brainstem and prefrontal cortex in patients with pain. Nonetheless, additional research is needed to explore the biological mechanisms through which PA may improve pain inhibitory function in patients with mTBI.

We have previously shown the presence of sensitization of the trigeminal pain system 1 to 2 weeks after injury in patients with patients,^3^ with enhanced PPTs of the head but not increased TS compared with matched controls. Contrary to our hypothesis, we did not find that PA behavior predicted the measures of sensitization in patients with mTBI. Research on the relationship between measures of sensitization and PA is mixed. Cross-sectional studies have demonstrated a relationship between heat TS and measures of moderate-to-vigorous PA in healthy adults^36,37^ but no relationship between mechanical TS and self-reported PA in healthy and chronic pain patients.^30,31^ Kroll et al.^18^ recently showed that an aerobic exercise intervention did not affect mechanical TS and PPTs of the head region in patients with migraine and coexisting tension-type headache. The relationship between PA and measures of sensitization may depend on a combination of factors including sample characteristics, the pain induction technique, the site of bodily application, and the method used to measure and categorize levels of PA.

MTBI is often accompanied by psychological distress,^32^ including high levels of pain catastrophizing.^3^ Pain catastrophizing refers to exaggerated and ruminating thoughts regarding true or anticipated pain. In general, a substantial amount of evidence supports the importance of pain catastrophizing in shaping pain-related experiences.^9^ Our data revealed that increased sitting at 1 to 2 weeks after injury predicted greater pain catastrophizing at 1 month after injury, even after controlling for headache pain. A recent diary study of older adults with osteoarthritis showed a reciprocal relationship between pain catastrophizing and sedentary behavior.^46^ Specifically, morning pain catastrophizing facilitated greater sedentary behavior later that day and the next day, which in turn exacerbated pain catastrophizing the following day. Taken together, the evidence suggests that greater sedentary behavior could worsen pain catastrophizing. It has been postulated that PA could be an effective strategy to distract attention from pain and decrease negative and ruminating thoughts about pain.^17^

Several limitations of this study need to be acknowledged. First, PA was assessed by a questionnaire rather than by objective methods. Subjective measures of PA can lead to overestimation and underestimation of the amount of PA reported, as answers depend on participant's memory. Thus, future research needs to confirm the current results with objective measures of PA. Second, we only assessed PA for 2 separate weeks in the 1 month after mTBI, which may not have been a representative of overall PA habits for each participant for the entire month. In addition, we were not able to collect PA data before the injury. It is possible that being physically active before a TBI facilitates better recovery. Third, all participants were recruited from the ED, and we excluded older adults, adolescents, and children. Thus, we do not know whether the current study's results would generalize to patients with mTBI not seen at the ED or different age groups.

In conclusion, our results contribute to the growing body of research suggesting that early moderate-to-light PA after mTBI may facilitate physiological and psychological recovery, including pain-related outcomes, after injury. We also provide some of the first longitudinal evidence showing that PA behavior can predict pain inhibitory capacity on the CPM test. Indeed, PA at 1 to 2 weeks after injury was not related to current pain catastrophizing or pain modulation capabilities but rather predicted future pain inhibitory function and catastrophizing 2 to 3 weeks later. Human and preclinical studies show impaired descending pain inhibition by the CNS and elevated pain catastrophizing early after mTBI.^3,19,38^ Therefore, based on our results, future research should explore whether moderate-to-light PA in the first weeks after mTBI could help restore deficient endogenous pain inhibitory capabilities or attenuate pain catastrophizing.

## Disclosures

There are not actual or potential conflicts of interest for any of the authors.
